# The Role of Clomipramine in Potentiating the Teratogenic Effects of Caffeine in Pregnant Rats: A Histopathological Study

**DOI:** 10.1155/2013/382434

**Published:** 2013-11-04

**Authors:** Vahid Nikoui, Sattar Ostadhadi, Nasrin Takzare, Seyyed Mohammad-Ali Nabavi, Mario Giorgi, Azam Bakhtiarian

**Affiliations:** ^1^Department of Pharmacology, School of Medicine, Tehran University of Medical Sciences, Keshavarz Boulevard, Tehran 1417613151, Iran; ^2^Department of Anatomy, School of Medicine, Tehran University of Medical Sciences, Tehran 1417613146, Iran; ^3^General Physician, School of Medicine, Tehran University of Medical Sciences, Tehran 1417613110, Iran; ^4^Department of Veterinary Sciences, University of Pisa, San Piero a Grado, 56122 Pisa, Italy

## Abstract

Since little is known about the teratogenic effects of clomipramine used concurrently with caffeine during the organogenesis period, the aim of this study was to test the teratogenic effects of a coadministration of caffeine and clomipramine on rat fetuses. We divided 42 pregnant rats into seven groups, randomly. The first group (control) received 0.5 mL of normal saline. Clomipramine was injected at 40 mg/kg and 80 mg/kg to the second and third groups, respectively. The fourth and fifth groups received caffeine in doses of 60 mg/kg and 120 mg/kg, respectively. The sixth group received a combination of 40 mg/kg clomipramine and 60 mg/kg caffeine, and the seventh group was given clomipramine and caffeine at 80 mg/kg and 120 mg/kg, respectively. The fetuses were removed on the 17th day of pregnancy and studied in terms of microscopic and macroscopic morphological features. Fetuses of rats receiving high doses of caffeine or combinations of caffeine and clomipramine showed a significant rate of cleft palate development, open eyelids, mortality, torsion anomalies, shrinkage of skin, and subcutaneous haemorrhage (*P* ≤ 0.001). This study concludes that caffeine in high doses or the simultaneous administration of caffeine and clomipramine leads to teratogenicity.

## 1. Introduction

For decades, it was postulated that the placenta acts as a barrier that defends the fetus from the adverse effects of drugs. The thalidomide tragedy overturned this conception, showing that use of some drugs during vital periods of fetal development result in serious limb defects and other organ anomalies [1]. Many drugs have been shown to affect pre- and/or postnatal development of the brain resulting in aberrant behaviour [2]. One of these drugs is clomipramine, a member of the tricyclic antidepressant group. This drug is prescribed for panic, depressive, and obsessive-compulsive disorders. After absorption following oral administration, it enters the brain and leads to reuptake inhibition of serotonin and norepinephrine in the synaptic cleft, resulting in increased concentrations of these two neurotransmitters in the synapse [3]. Clomipramine enters fetal blood via the placenta due to its highly lipophilic properties [4].

Caffeine is a natural alkaloid compound found in coffee, tea, and cola drinks; it is metabolized by liver cytochrome P_450_ enzymes. This agent is easily absorbed from the gut and readily passes through placenta, so fetal and maternal plasma concentrations reach an equilibrium [5]. Following the disaster caused by thalidomide, an antivomiting agent which when administered to pregnant women caused limb deformities in new born infants, the teratogenic properties of drugs were considered in a new light [6]. It has been established that caffeine consumption by pregnant women can have adverse effects on the fetus and as clomipramine inhibits the metabolism of caffeine [7], it is possible that clomipramine may increase the teratogenicity of caffeine. Many articles about the likely effects of antidepressants on the fetus have been published [8–10], but adequate studies in humans are not available [11]. Teratogenic effects of clomipramine have not been observed via the oral (mice and rats), subcutaneous (mice and rats), and intravenous (mice and rabbits) routes of administration [12]. In pregnant women, prescription of low amounts of clomipramine three times daily has had no teratogenic effects [13]. Researchers studied the effect of caffeine on pregnant women from the eighth to the twelfth week of pregnancy and results showed that even small amounts of caffeine had negative effects on fetal growth. They concluded that a daily intake of 200 mg of caffeine causes a significant increase in the abortion rate [14]. Caffeine and clomipramine are classified in pregnancy category C. The most critical time during pregnancy is the embryonic period [15]. Therefore, caution in prescription is necessary, particularly at this time. There is evidence that coadministration of caffeine and clomipramine has a potentiating effect on caffeine toxicity [7]; so the aim of this study was to investigate the effects of simultaneous administration of different doses of these two drugs on fetal development in pregnant rats.

## 2. Materials and Methods

### 2.1. Animals

As the animal model, the Wistar-Albino strain of the laboratory rat inbred for 96 generations by brother sister litter-male mate mating was used. This strain was used for many years in our laboratory in Tehran University of Medical Science (2006-2007) and has never shown a prediction for spontaneous maternal defects. Healthy adult female and male rats with an average age of approximately three month and weighing 300–350 grams were randomly selected. They were kept in a controlled room (temperature, 20 to 25°C, humidity, 70% to 80%, exposed to 12 h of daylight). The rats were fed with standard rat food and tap water until experimentation. Limitation of food and water was not applied to the animals that were put into their cages after the experiments. After mating, and ensuring successful conception, pregnant rats were divided into seven groups (*n* = 6). All experiments were conducted in Tehran University of Medical Sciences according to the recommendations of the ethics committee on animal's experimentation of medical school.

### 2.2. Drugs

In all groups, predetermined doses of drugs were daily injected intraperitoneally between the eighth and fifteenth day of pregnancy. The first group was used as the control and received one mL of normal saline. In the second and third groups, clomipramine was injected in doses of 40 mg/kg and 80 mg/kg, respectively. Caffeine was injected at 60 mg/kg and 120 mg/kg to the fourth and fifth groups, respectively. Rats in group six received 40 mg/kg clomipramine and 60 mg/kg caffeine. Finally, the seventh group was administrated with clomipramine and caffeine at 80 mg/kg and 120 mg/kg, respectively. Drugs were purchased from Sigma-aldrich Company, USA. On the 17th day of pregnancy, the animals were anesthetized via inhalation of high concentrations of chloroform and the fetuses were removed by caesarean section.

### 2.3. Macroscopic and Microscopic Studies

They were then examined for macroscopic abnormalities. Histopathological slides from fetuses were also prepared. After hematoxylin and eosin staining, any microscopic changes in fetuses were noted using an optical microscope. Fetuses with abnormal body shape (non-C-shaped), subcutaneous hemorrhage, skin shrinkage, bent limbs, unilateral or bilateral cleft palates, and nonfused eyelids were considered abnormal [16].

### 2.4. Statistical Analysis

Data were analysed using statistical software SigmaPlot version 11. Chi-square test was used to ascertain the significance of variations between frequencies of abnormal fetuses in different groups. Differences were considered significant at *P* ≤ 0.001.

## 3. Results

**Figure 1:**
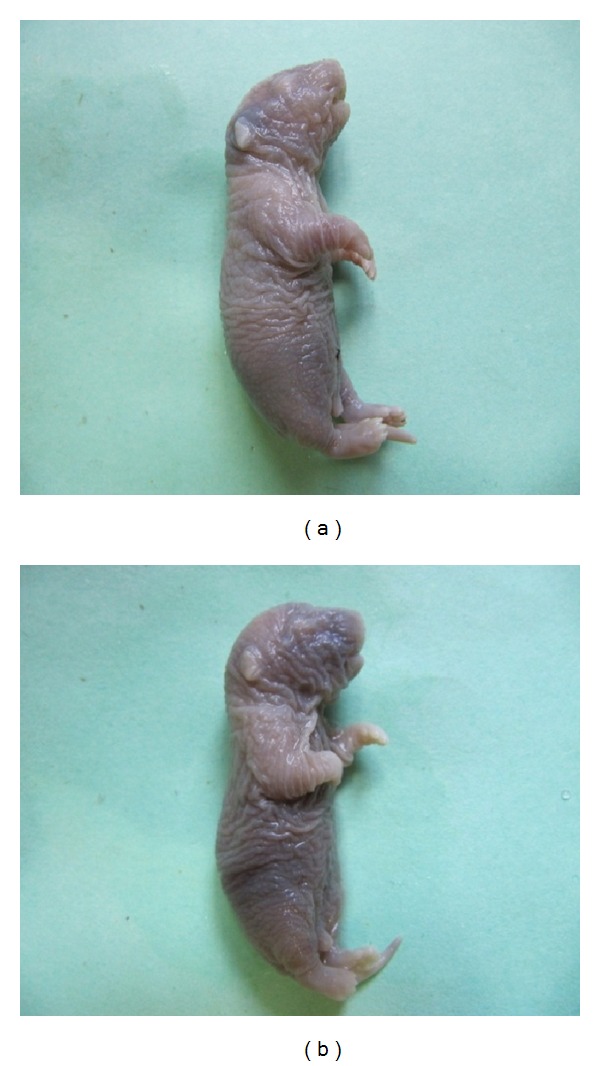
Macroscopic view of 17-day-fetuses from control group (a) and the group that received a combination of clomipramine and caffeine in high doses (b). In the control group fetus, the muzzle is formed normally. The body is C-shaped and eyes, ears, and upper and lower extremities are in their normal locations. The skin is taut (a). In the fetus from the group treated with clomipramine and caffeine in high doses, the body is not fully C-shaped, the forelimbs are bent, and corrugated skin and areas of subcutaneous hemorrhage are obvious (b).

**Figure 2:**
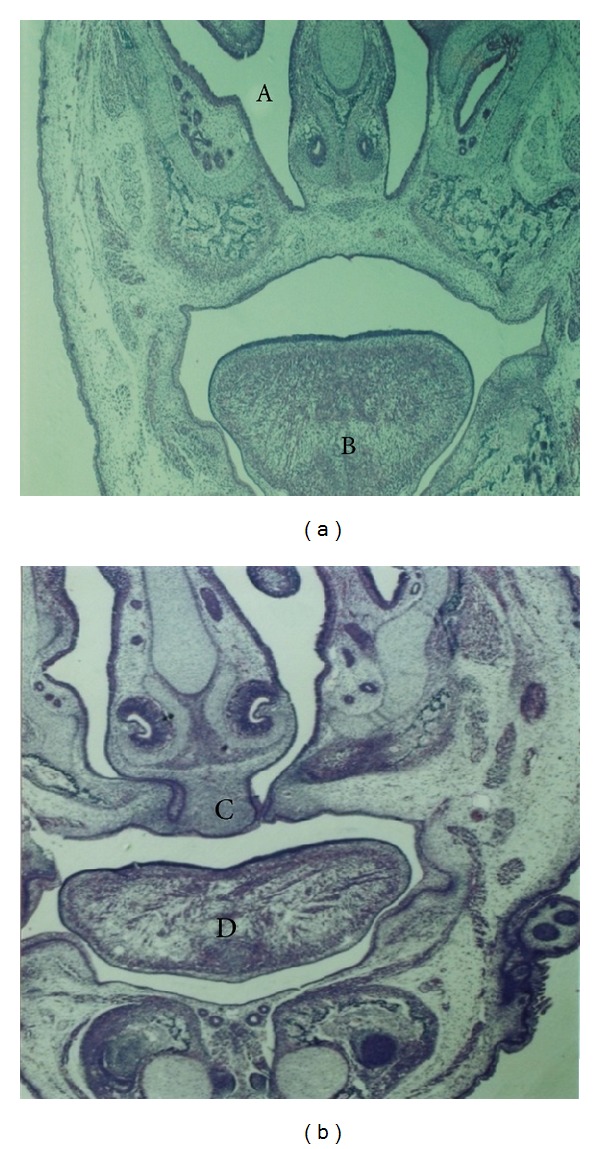
Histopathological slides of the frontal section of the head, 17-day-fetuses in the control group (a) and the group that received a combination of clomipramine and caffeine in high doses (b) (H&E staining, 4x). In the control group (a), the nasal septum is attached to the roof of the mouth and nostrils (A) are completely separated from the oral cavity. Tongue (B) is shown to be perfectly in the mouth and located in its normal place. In the fetus from the group treated with clomipramine and caffeine in high doses (b), there is bilateral clefting of the palate (C) and tongue inside the mouth is flat (D).

**Figure 3:**
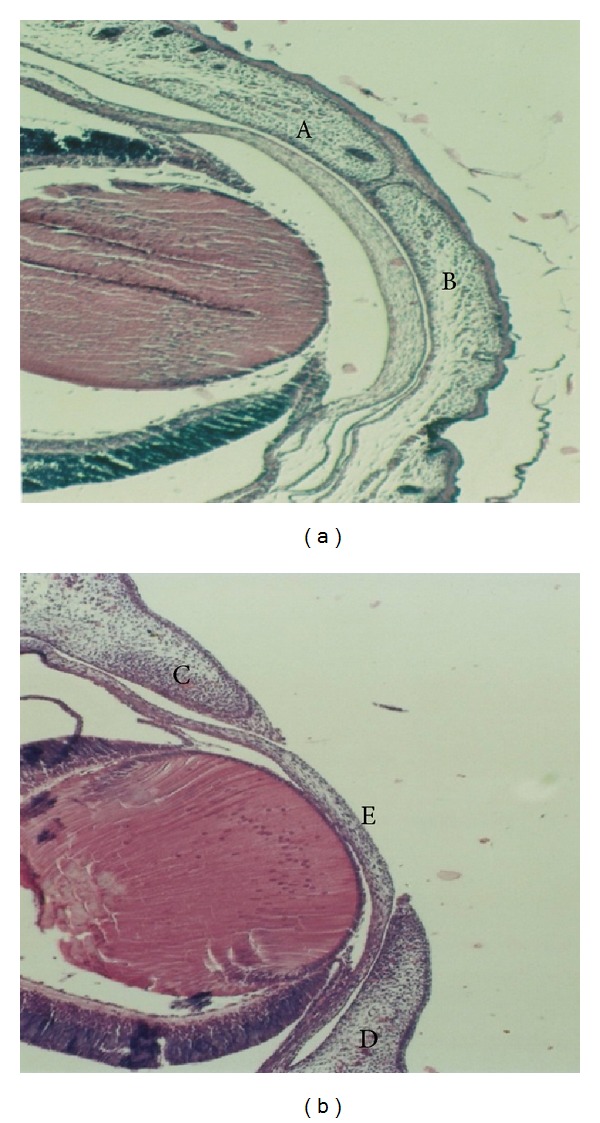
Histopathological slides of the eyes from 17 days fetuses in control group (a) and the group received a combination of clomipramine and caffeine in high doses (b) (H&E staining, 10x). In control group fetus (a), the upper (A) and lower (B) eyelids are joined completely. In the fetus from the group treated with clomipramine and caffeine in high doses (b), the upper (C) and lower (D) eyelids were separated completely and the cornea (E) is exposed.

No abnormal development of the fetal body or limbs was observed in the control or low dose (40 mg/kg) clomipramine group. In contrast, several fetuses in the remaining groups demonstrated abnormal development. Likewise, neither shrunk skin nor subcutaneous bleeding was noted in the control or low dose clomipramine group, however in the other groups, multiple fetuses had obvious skin wrinkling and local bleeding under the skin. Abnormalities in other tissues such as the ear, neck, and tail were not observed in the control, low dose clomipramine, high dose clomipramine (80 mg/kg), and low dose (60 mg/kg) caffeine groups, however in the high dose (120 mg/kg) caffeine, combination of clomipramine and caffeine in low doses, and combination of clomipramine and caffeine in high doses groups, several anomalies were seen. The chi-square statistical analysis showed that the differences in the number of apparent anomalies between the control group and high dose caffeine, combination of clomipramine and caffeine in low doses, and combination of clomipramine and caffeine in high doses groups are significant (*P* ≤ 0.001). We also found a significant difference in the number of apparent anomalies between the high dose caffeine group as compared to the combination of clomipramine and caffeine in low doses and combination of clomipramine and caffeine in high doses groups (*P* ≤ 0.001). In the morphological exam, 17-day-old fetuses of the control group had formed their normal C-shaped body with normal extremities. Frontal and maxillary appendages were fused together and the muzzle was formed normally. The lips and mouth were located in the normal position, as were eyes and ears, eyelids were fused together (Figure 1(a)). In the group that received a combination of clomipramine and caffeine in high doses (80 mg/kg and 120 mg/kg, resp.), fetuses had an abnormal body shape and short limbs. In some samples the jaw, nose, ears, and lips were not in their normal positions (Figure 1(b)). Histopathological slides from frontal sections of control group showed that the wall of the nose (nasal septum) was located in the middle of the nasal cavity and was connected to the roof of the mouth. The oral cavity was completely isolated from the nasal cavity and tongue was found to be located in its normal place, the mouth (Figure 2(a)). Eyelids were fused together and the cellular layers of eyeball were normal (Figure 3(a)). Microscopic slides of frontal skull sections in the group that received a combination of clomipramine and caffeine in high doses revealed unilateral and bilateral cleft palates in some samples (Figure 2(b)). In addition, eyelids were not fused together in this group (Figure 3(b)).

## 4. Discussion

While pregnant, there are two main areas to consider when taking pharmaceuticals or consuming food or drinks that contain other forms of drugs. The first is potential adverse effects on fetus; the second is any effects that pregnancy may have on the clinical pharmacokinetics of a drug. The thalidomide disaster emphatically highlighted the importance of investigating and considering the teratogenicity of drugs that may be administered during pregnancy [6]. Although this awareness had led to a decrease in the instances of teratogenic drugs being administered to pregnant women, there is still insufficient research being performed in this field. We may not have fully mitigated the risk of a similar episode occurring in the future. Drugs with low teratogenic potential do not result in adverse effects in the majority of pregnant women; however, in a small number of pregnancies, they may still cause teratogenesis. 

There is a high reported prevalence of obsessive-compulsive disorder development and depression during the pregnancy period. For this reason, there is an increased probability that pregnant women might be prescribed clomipramine and be taking this, along with caffeine-containing drugs and food. In the present study, 17-day-fetuses from control and experimental groups were examined for macroscopic and microscopic pathology. There is 120 mg of caffeine in a 300 mL cup of coffee. The therapeutic dose of caffeine for treatment of postdural-puncture headache (PDPH) in humans is 7 mg/kg twice daily, while we used 60 and 120 mg/kg of caffeine in rats, and this difference is a result of metabolism dissimilarities between humans and rats. The threshold teratogenic dose of caffeine after an acute injection is 80–100 mg/kg in rats. In rats, malformations are infrequently observed after single daily doses less than 80–100 mg/kg and almost never observed at doses less than 50 mg/kg/day [17]. Weng et al. (2008) investigated the effects of caffeine on pregnant women in the eighth to twelfth week of pregnancy. They showed that small amounts of caffeine affected fetal growth and daily intake of 200 mg of caffeine significantly increased the rate of spontaneous abortion [14]. No teratogenic effects of clomipramine were observed in studies carried out in rats and mice at doses up to 100 mg/kg/day, which is 24-fold the maximum recommended human daily dose on a mg/kg basis and four times (rats) and two times (mice) the recommended dose on a mg/m^2^ basis. Minor nonspecific fetotoxic effects are seen in baby rats treated with 50 and 100 mg/kg and in babies of mice treated with 100 mg/kg [18]. In the present study, clomipramine administration alone also failed to induce teratogenic effects at doses of 40 mg/kg and 80 mg/kg. Although the teratogenic effect of caffeine was low at a dose of 60 mg/kg, the coadministration of clomipramine and caffeine at low doses led to significant teratogenic effects (Figure 1(b)). The metabolism of drugs is catalyzed by selective cytochrome P_450_ (CYP) isoenzymes. Caffeine and clomipramine are substrates for liver CYP1A2 enzyme, and approximately 90% of caffeine metabolism occurs via CYP1A2 [19]. Among the investigated antidepressants, the tricyclic antidepressant drugs imipramine, clomipramine, and desipramine and the selective serotonin reuptake inhibitor (SSRI) sertraline are the most powerful inhibitors of rat CYP1A2. The effect of antidepressants is approximately ten times weaker in rats than in humans, as a result of species differences in CYP1A2 structure and function [7]. Therefore, it is postulated that simultaneous administration of these drugs in humans is likely to result in more adverse effects. 

On the other hand, CYP1A2 and CYP2C19 activity is decreased during pregnancy, indicating that a dose reduction may be required to minimize the potential toxicity of their substrates [20]. 

## 5. Study Limitations

Metabolism differences between rats and humans, and so dosage dissimilarities among various species can be considered as a limitation of the present study.

## 6. Conclusions

Although the present research has been carried out in rats and caution should be used in extrapolating this data to human beings, it might be concluded that because of the potential teratogenic effects of caffeine, low activity of CYP1A2 during pregnancy, and inhibitory effect of clomipramine on CYP1A2 activity, the dose of caffeine recommended as safe during pregnancy should be decreased for women simultaneously taking antidepressants, especially clomipramine.
